# SGK1 mediates TGF-β1-induced epithelial-mesenchymal transition and fibrosis in experimental bronchiolitis obliterans

**DOI:** 10.1016/j.isci.2026.117961

**Published:** 2026-10-19

**Authors:** Hongwei Li, Li Zhao, Shihui Xu, Sixiang Tang, Junxing Lin, Yingjie Hu, Xiaowen Chen, Chengyu Lu, Dehui Chen

**Affiliations:** 1 Department of Pediatrics, The First Affiliated Hospital of Guangzhou Medical University, Guangzhou, Guangdong, China; 2 Department of Pediatrics, Guangzhou Medical University, Guangzhou, Guangdong, China

**Keywords:** bronchiolitis obliterans, SGK1, epithelial-mesenchymal transition, fibrosis

## Abstract

Bronchiolitis obliterans (BO) represents a major complication limiting long-term survival after lung transplantation, with epithelial-mesenchymal transition (EMT) serving as a core pathogenic mechanism. The TGF-β1 signaling pathway plays a critical role in EMT induction and can activate serum/glucocorticoid-regulated kinase 1 (SGK1) expression. While SGK1 is upregulated in various fibrotic diseases, its functional significance in BO remains undefined. To elucidate SGK1’s role in post-transplant BO fibrosis and its interaction with the TGF-β1 pathway, we established a murine heterotopic tracheal transplantation model. Histopathological evaluation and RNA sequencing demonstrated significant SGK1 upregulation in BO lesions by day 28. *In vitro*, both pharmacological inhibition (EMD638683) and genetic knockdown of SGK1 (siRNA) attenuated TGF-β1-induced EMT in the human small airway epithelial cell line (HSAECs), while concurrently suppressing the expression of extracellular matrix genes (COL1 and FN1). Furthermore, SGK1 inhibition reduced TGF-β1-induced SMAD2/3 phosphorylation. Therapeutic administration of EMD638683, initiated after disease establishment, significantly ameliorated BO pathology. These findings demonstrate that SGK1 promotes BO-associated fibrosis through TGF-β1-mediated EMT and ECM deposition, identifying SGK1 as a potential therapeutic target for post-transplant BO intervention.

## Highlights

• Reveal the regulatory role of SGK1 in post-transplant bronchiolitis obliterans
• Find that SGK1 expression is up-regulated in the mouse model on day 28
• Find that EMD638683 can alleviate the fibrosis process of bronchiolitis obliterans
• Suggest that SGK1 is a potential target for bronchiolitis obliterans intervention

## Graphical abstract

**Figure:**
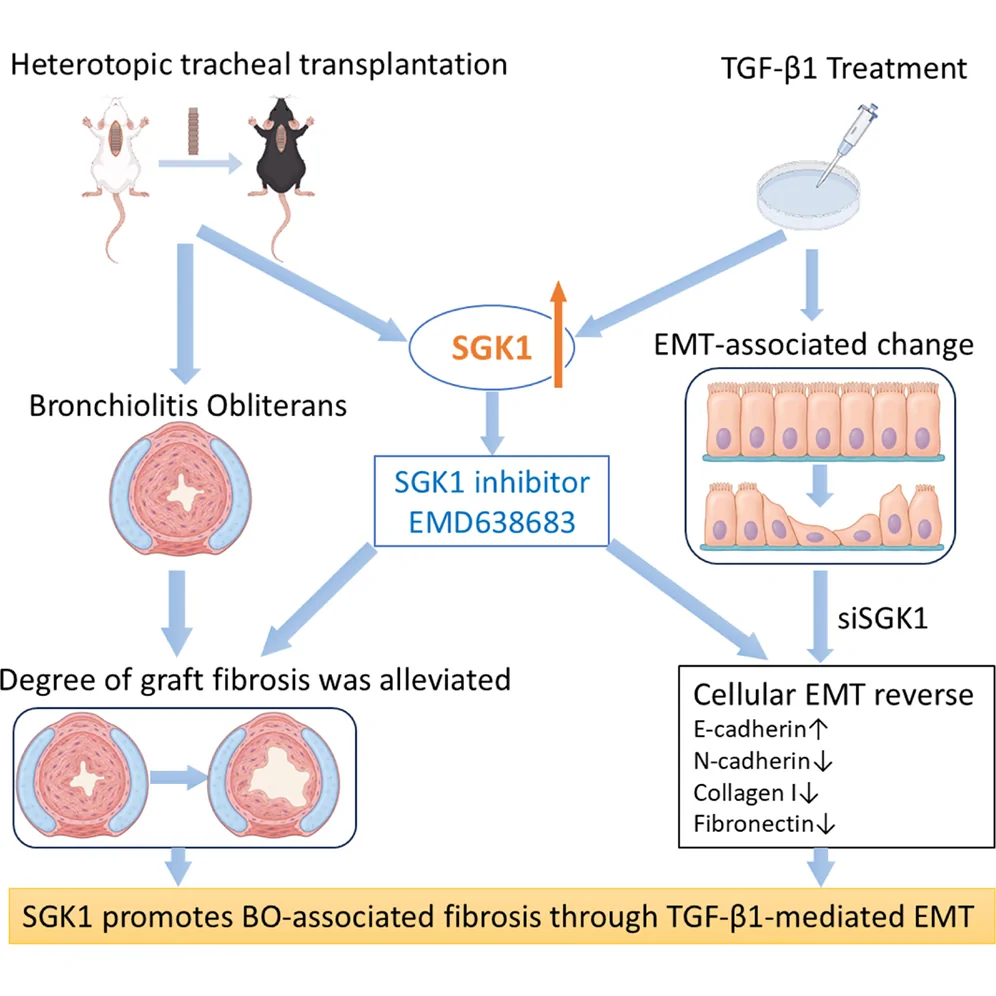

## Teaser

Molecular biology; Medical biotechnology

## Introduction

Lung transplantation is currently one of the only effective treatments for end-stage pulmonary diseases such as idiopathic pulmonary fibrosis, chronic obstructive pulmonary disease, and cystic fibrosis.^1^ Bronchiolitis obliterans (BO) constitutes a serious complication following lung transplantation, characterized by progressive fibrosis and luminal narrowing of small airways, ultimately leading to airway obstruction.^2^^,^^3^ The pathogenesis of BO involves epithelial-mesenchymal transition (EMT) of small airway epithelial cells, wherein these cells lose epithelial characteristics (cell polarity, intercellular junctions, basement membrane attachment) and acquire mesenchymal phenotypes (enhanced migration, extracellular matrix synthesis).^4^

Transforming growth factor β1 (TGF-β1) serves as a potent inducer of EMT in small airway epithelial cells,^5^ and promotes the transdifferentiation of these cells into myofibroblasts under the stimulation of TGF-β1, which are the main collagen-producing cells of BO.^6^ Therefore, inhibition of TGF-β1 signaling is regarded as a potential strategy for the treatment of BO.

TGF-β1 promotes inflammation and fibrosis through transcriptional activation of serum/glucocorticoid-regulated kinase 1 (SGK1) mRNA.^7^ SGK1, a serine/threonine protein kinase, regulates diverse physiological processes and exhibits widespread tissue distribution.^8^ While SGK1 demonstrates low basal expression under resting conditions, it responds robustly to various stimuli and functions as a key cellular stress mediator.^9^ Pathological SGK1 upregulation occurs in multiple fibrotic conditions including pulmonary fibrosis, diabetic nephropathy, hepatic cirrhosis, and cardiac fibrosis.^10^^,^^11^^,^^12^ However, SGK1’s role in BO pathogenesis remains unexplored.

EMD638683, a highly selective SGK1 inhibitor, has demonstrated efficacy in various fibrotic disease models. It prevents angiotensin II-induced cardiac inflammation and fibrosis by blocking NOD-like receptor family pyrin domain containing 3 (NLRP3) inflammasome activation^7^^,^^13^^,^^14^^,^^15^^,^^16^^,^^17^; EMD638683 administration inhibits macrophage infiltration and pulmonary artery smooth muscle cell proliferation in monocrotaline (MCT)-induced pulmonary arterial hypertension (PAH) rats^18^; and modulates epithelial sodium channel (ENaC)-mediated alveolar fluid clearance in acute lung injury models.^19^ Additionally, EMD638683 suppresses colon tumor growth.^20^ Nevertheless, its potential therapeutic effect in BO remains uninvestigated.

This study employed murine heterotopic tracheal transplantation and TGF-β1-stimulated human small airway epithelial cell models to investigate SGK1’s regulatory mechanism in TGF-β1-mediated BO fibrosis. Our findings establish SGK1 as a critical mediator of TGF-β1-driven EMT and fibrosis in BO, revealing its potential as a therapeutic target.

## Results

### Characterization of the BO mouse model reveals progressive fibrosis and ECM remodeling

The control group and BO group were constructed using the modeling principles of allotopic and heterotopic transplantation, respectively, and samples were collected on days 7, 14, and 28 for analysis (Figure 1A). Compared with the control group, the transplanted tracheas in BO mice on days 7, 14, and 28 showed higher rates of epithelial cell loss, lumen occlusion, inflammatory cell infiltration, structural destruction and deformation of the tracheal cartilage ring, higher relative collagen content, higher percentages of COL1-and FN1-positive immunohistochemical staining, and a lower percentage of E-cadherin-positive immunohistochemical staining. The pathological findings in the transplanted tracheas of BO mice became more typical as the duration of transplantation increased. On day 28, statistically significant differences were observed between the BO and control groups in epithelial cell loss (Figure 1C), relative collagen content (Figure 1E), and immunohistochemical staining intensities for COL1 (Figure 1F), FN1 (Figure 1G), and E-cadherin (Figure 1H). Furthermore, the lumen occlusion rate differed significantly between BO mice on day 28 and those on day 7 (Figure 1D).

**Figure 1:**
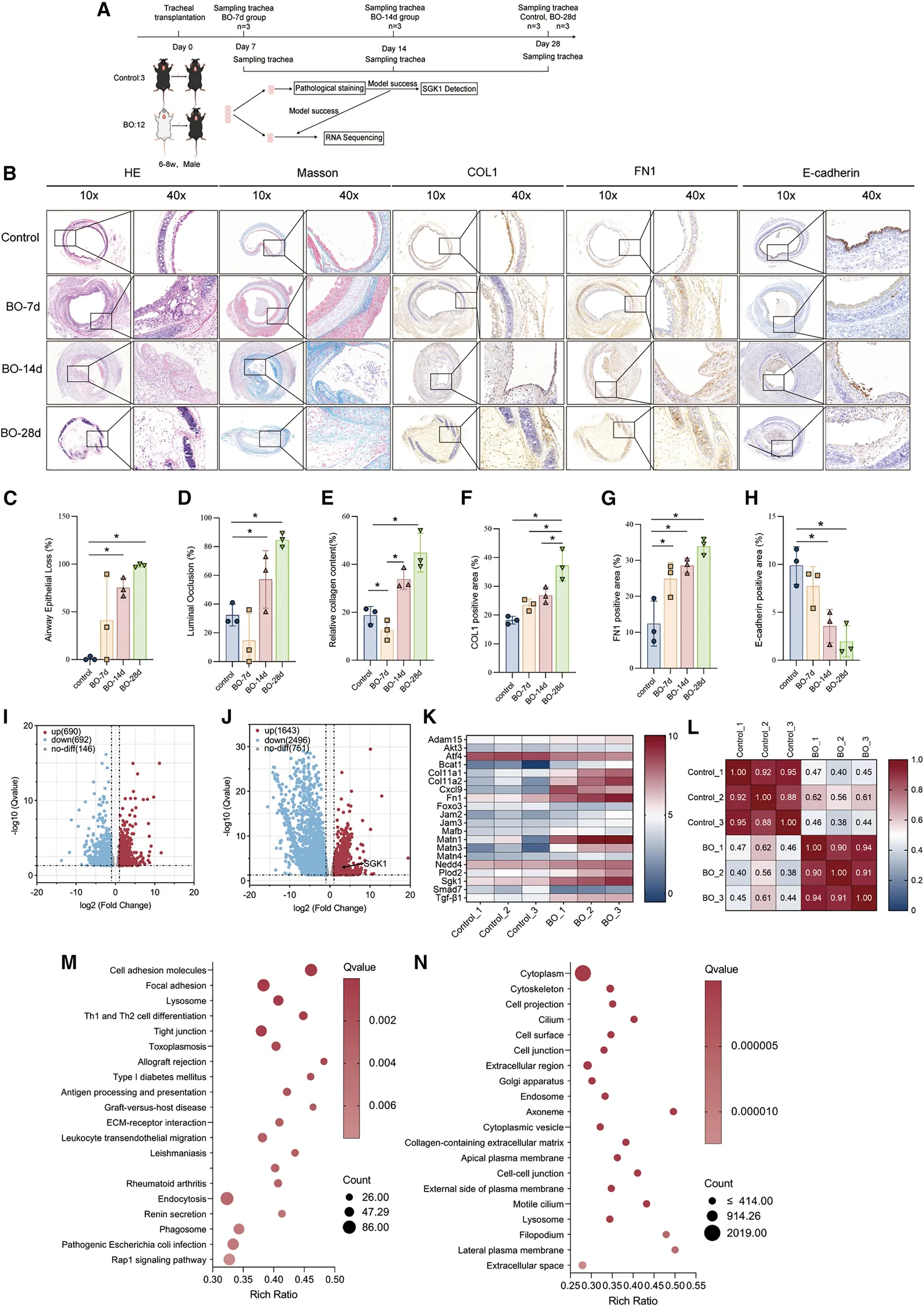
Typical pathology of the BO mouse model on day 28 Differentially expressed genes were significantly enriched in graft rejection and ECM pathways. (A) Experimental flowchart of mouse heterotopic tracheal transplantation model. (B) Representative images of HE, Masson, and immunohistochemical staining of the control group, 7-day model group (BO-7d), 14-day model group (BO-14d), and 28-day model group (BO-28d). (C) Epithelial cell loss rate in the four mouse groups. (D) Lumen occlusion rate in the four mouse groups. (E) Relative collagen content in the four mouse groups. (F–H) Percentage of immunohistochemically positive area for collagen type I (COL1) (F), fibronectin 1 (FN1) (G), and E-cadherin (H) in the four mouse groups. (I) Volcano plot (BO-7d vs. control). (J) Volcano plot (BO-28d vs. control). (K) Heatmap (BO-28d vs. control). (L) Sample correlation coefficient heatmap (BO-28d vs. control). (M) KEGG pathway enrichment bubble plot (BO-28d vs. control). (N) GO cellular component enrichment bubble plot (BO-28d vs. control). Pathological diagram: 10×: scale bars, 100 μm, 40×: scale bars, 20 μm. *n* = 3. The data in (C–H) are presented as mean ± SD. ∗: *p* < 0.05, based on one-way ANOVA with Tukey’s post-hoc test. Volcano plot: differentially expressed genes required |log_2_(fold change)| > 1 and *p < 0.05*. The KEGG pathway enrichment bubble plot and GO cellular component enrichment bubble plot showed the most significantly enriched 20 signaling pathways and cellular components, respectively. Functions with *Q-value < 0.05* were considered significantly enriched.

In the samples of BO mice tested, there were 146 genes with no difference between the BO group and the control group on the 7th day, and there were 1382 differentially expressed genes, including 690 up-regulated differentially expressed genes and 692 down-regulated differentially expressed genes, and no up-regulated expression of SGK1 was observed. The gene distribution was shown in the volcano diagram (Figure 1I). On the 28th day, there were 751 genes with no difference between the BO group and the control group, and there were 4139 differentially expressed genes, of which 1643 genes were up-regulated and 2496 genes were down-regulated. The expression of SGK1 was up-regulated, and the gene distribution was shown in the volcano map (Figure 1J) and heatmap (Figure 1K).

Combined with the results of the pathological section, volcano plot, and heatmap, it was shown that BO pathological changes could occur on the 7th and 28th day in the BO group, but the pathological changes of BO mice on the 28th day were more typical, suggesting that the BO mouse model on the 28th day was more stable than that on the 7th day and more suitable for the in-depth mechanism study of BO. Subsequently, in order to ensure the credibility of the experimental results, only the expression of the BO group on day 28 and the control group on day 28 were analyzed in the subsequent study.

The correlation coefficient map of samples between the BO group and the control group on day 28 further showed that the gene expression differences between the two groups were large, and the correlation coefficient between the samples of the two groups was low (Figure 1L). KEGG Pathway enrichment analysis showed that the differentially expressed genes in the BO group on day 28 were significantly enriched in Th1 and Th2 cell differentiation, graft rejection, and ECM-receptor interaction compared with the control group (Figure 1M). The results of GO Cellular enrichment analysis showed that the differentially expressed genes were significantly enriched in extracellular matrix (ECM), such as collagen and other cellular components, in BO mice on day 28 compared with control mice (Figure 1N).

### SGK1 expression is increased in mouse tracheal allografts and TGF-β1-induced human myofibroblasts

To further verify the expression of SGK1 in the transplanted trachea of BO mice, SGK1 immunohistochemical staining was performed on the transplanted trachea of BO mice on day 28. The results of SGK1 immunohistochemistry showed that compared with the control group, the expression of SGK1 in the BO group was significantly increased on the 28th day, and the difference was statistically significant (Figures 2A and 2B).

**Figure 2:**
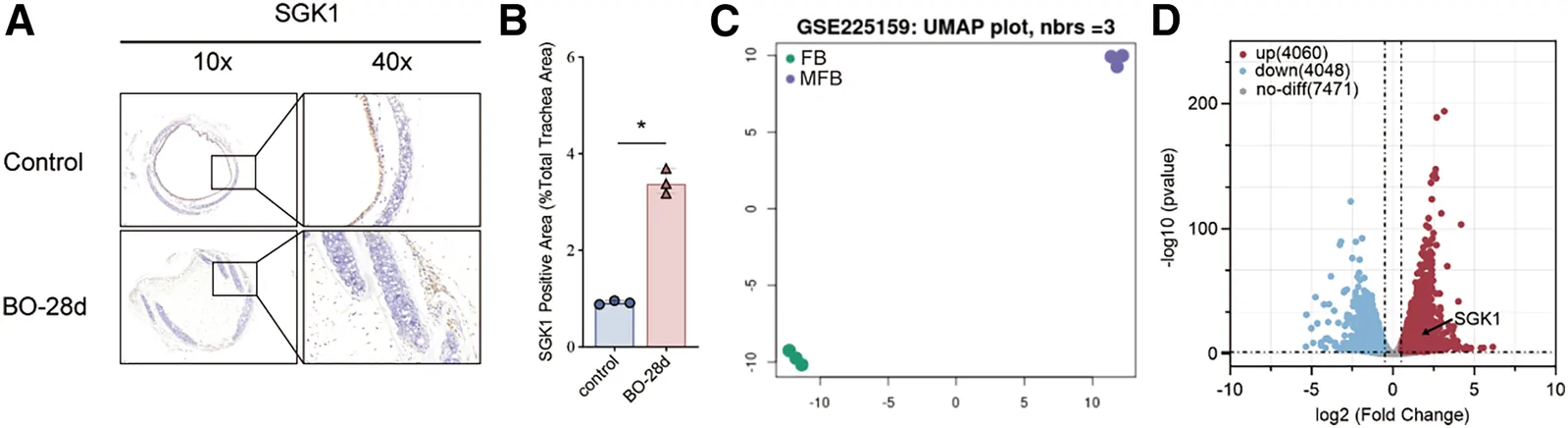
SGK1 expression is increased in mouse tracheal allografts and TGF-β1-induced human myofibroblasts (A) Representative immunohistochemical staining for SGK1 in syngeneic control and allogeneic tracheal grafts on day 28 after transplantation. (B) Quantification of SGK1-positive area in the graft sections. (C) UMAP visualization of untreated MRC-5 lung fibroblasts (FB) and TGF-β1-induced myofibroblasts (MFB) from GSE225159, with three samples per group. (D) Volcano plot showing differential gene expression in MFB relative to FB, with SGK1 indicated. 10×: 100 μm, 40×: 20 μm. *n* = 3, ∗:*p* < 0.05.

To examine SGK1 expression in an additional human cellular model of fibrosis, we reanalyzed the GSE225159 dataset.^21^ This analysis compared untreated MRC-5 lung fibroblasts (FB) with TGF-β1-induced myofibroblasts (MFB), with three samples per group. UMAP visualization showed separation between the FB and MFB samples (Figure 2C). Differential expression analysis identified 8,108 differentially expressed genes, including 4,060 upregulated and 4,048 downregulated genes in MFB relative to FB. SGK1 was among the upregulated genes (Figure 2D), supporting its association with TGF-β1-induced fibroblast-to-myofibroblast transition.

### EMD638683 significantly reduced the pathological indicators and the expression of SGK1, COL1, and FN1 in BO mice

The model of allotopic transplantation was used to establish the control group, and the model of heterotopic transplantation was used to establish the BO group and the Inhibitor group. The control and BO groups received the vehicle solution of the SGK1 inhibitor via intragastric gavage daily, starting two days before modeling; the inhibitor group received the SGK1 inhibitor working solution via oral gavage daily, starting two days before modeling. HE and IHC staining results showed that compared with the control group, the BO group had increased epithelial cell loss rate, lumen occlusion rate, and immunohistochemical positive area percentages of SGK1, COL1, and FN1. At the same time, the degree of inflammatory cell infiltration, damage, and deformation of the tracheal cartilage ring also increased. Compared with the BO group, the lumen occlusion rate and the percentages of SGK1, COL1, and FN1 immunohistochemically positive areas in the Inhibitor group decreased, and the differences were statistically significant (*p* < 0.05). At the same time, the degree of inflammatory cell infiltration and the degree of structural damage and deformation of the tracheal cartilage ring also decreased (Figures 3B–3G).

**Figure 3:**
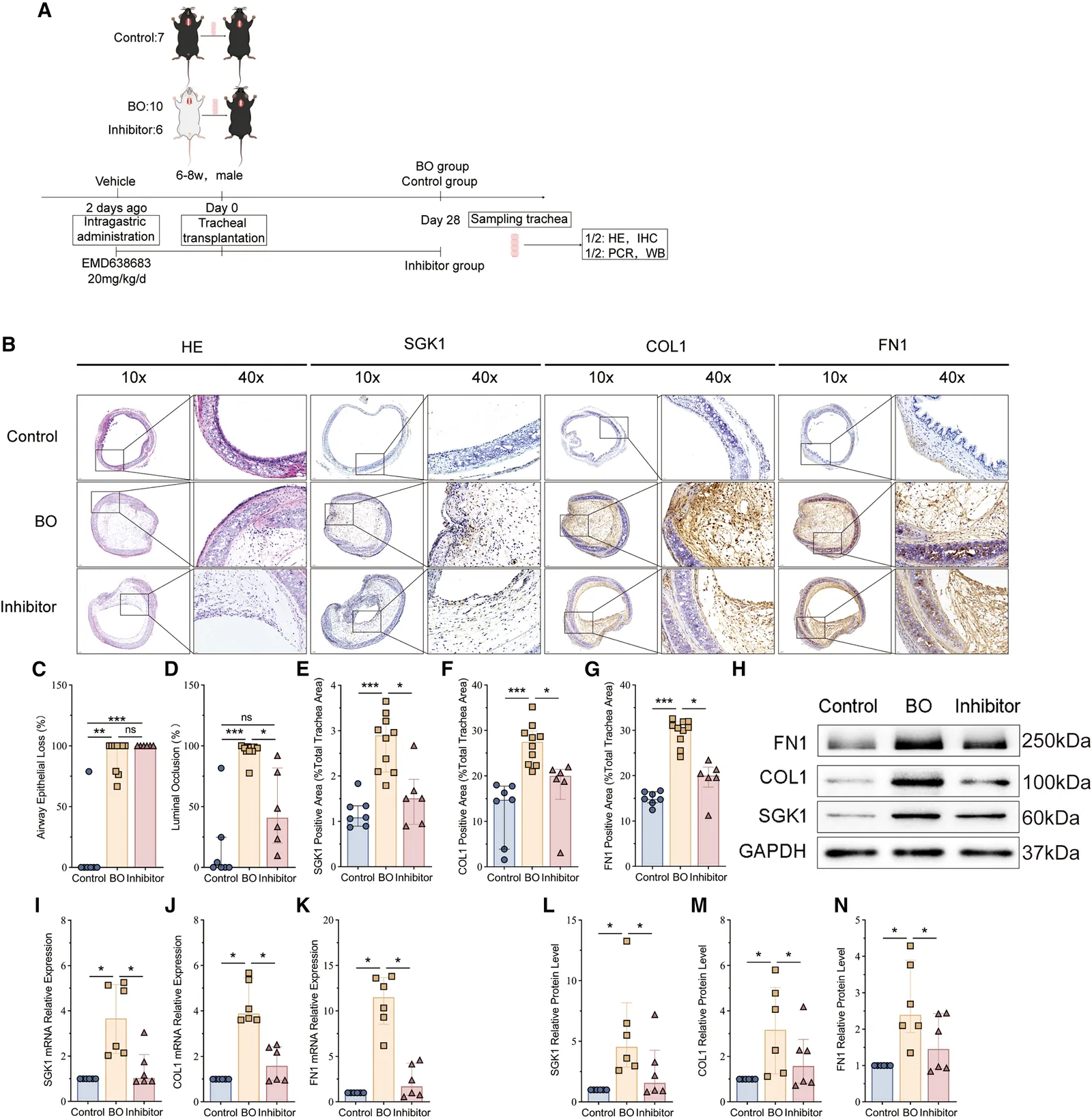
EMD638683 (20 mg/kg/d) significantly reduced the pathological indicators and the expression of SGK1, COL1 and FN1 in BO mice (A) Experimental flow chart of tracheal transplantation model in mice. (B) Representative HE and immunohistochemical staining sections of SGK1, COL1 and FN1 in the three mouse groups. (C) Epithelial cell loss rate in the three mouse groups. (D) Lumen occlusion rate in the three mouse groups. (E–G) SGK1 (E), COL1 (F), FN1 (G) positive area percentage of the three groups of mice; (H) WB trend plots of the three groups of mice. (I–K) SGK1(I), COL1(J) and FN1(K) mRNA levels in the three groups. (L–N) Relative protein expression levels of SGK1(L), COL1(M), and FN1(N) in the three groups of mice. Pathological diagram: 10×: scale bars, 100 μm, 40×: scale bars, 20 μm. For histological analyses (C–G), *n* = 7 control mice, 10 BO mice, and 6 EMD638683-treated mice. For RT-qPCR analyses (I–K) and western blot quantification (L–N), *n* = 6 mice per group. Each data point represents one mouse. Data are presented as mean ± [SD or SEM]. Statistical comparisons were performed using one-way ANOVA followed by Tukey’s test. ∗*p* < 0.05, ∗∗*p* < 0.01, and ∗∗∗*p* < 0.001; ns, not significant.

The results of PCR and WB showed that the mRNA and protein expression levels of SGK1, COL1, and FN1 in the BO group were higher than those in the control group , and the expression levels of SGK1, COL1, and FN1 in the Inhibitor group were lower than those in the BO group. The differences were statistically significant (*p* < 0.05) (Figures 3H–3N).

Next, we adjusted the drug administration timing to post-heterotopic tracheal transplantation in mice. The control group was established using a syngeneic heterotopic transplantation model, while the BO and inhibitor groups were generated via an allogeneic heterotopic transplantation model. Notably, mice in the control and BO groups received daily intragastric gavage of the SGK1 inhibitor vehicle starting on post-modeling day 7, whereas the inhibitor group received daily intragastric gavage of the SGK1 inhibitor working solution from the same time point.

HE and Masson staining revealed that, compared with the control group, the BO group exhibited significantly increased epithelial cell loss rate (Figure 4C), lumen occlusion rate (Figure 4D), and relative collagen content (Figure 4E) (all *p* < 0.05). In contrast, the inhibitor group showed significantly reduced lumen occlusion rate (Figure 4D) and relative collagen content (Figure 4E) compared with the BO group (both *p* < 0.05), indicating that SGK1 inhibitor EMD638683 treatment effectively alleviates post-transplant lumen stenosis induced by collagen deposition.

**Figure 4:**
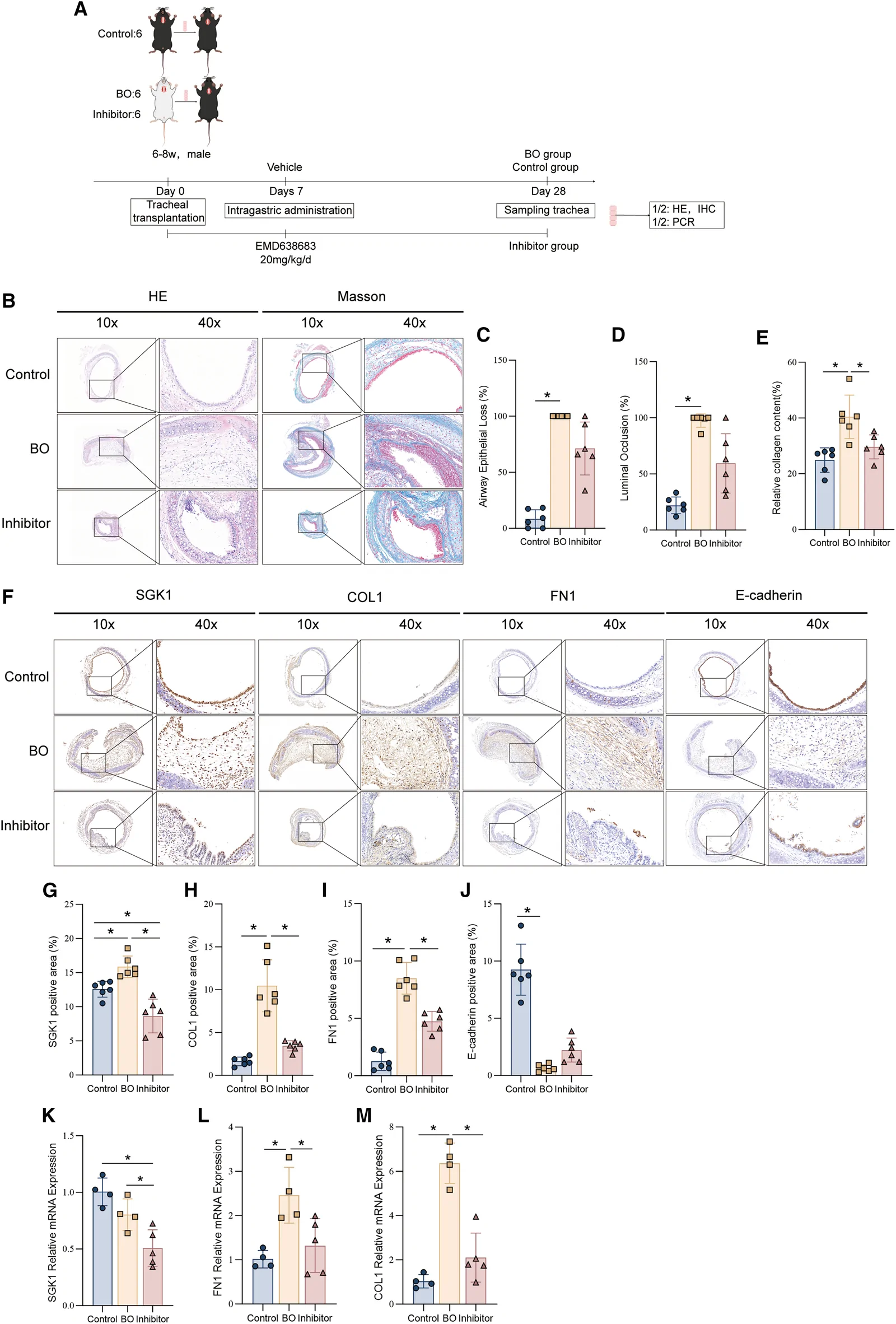
EMD638683 (20 mg/kg/d) treatment effectively alleviates post-transplant lumen stenosis induced by collagen deposition (A) Experimental flowchart of tracheal transplantation model in mice. (B) Representative images of HE and Masson staining of the control group, BO group, and inhibitor group. (C) Epithelial cell loss rate in the three mouse groups. (D) Lumen occlusion rate in the three mouse groups. (E) Relative collagen content in the three mouse groups. (F) Representative images of immunohistochemical staining in the three mouse groups. (G–J) Percentage of immunohistochemically positive area for SGK1 (G), COL1 (H), FN1 (I), and E-cadherin (J) in the three mouse groups. (K–M) SGK1 (K), FN1 (L) and COL1 (M) relative mRNA expression levels in the three mouse groups. Pathological diagram: 10×: scale bars, 100 μm, 40×: scale bars, 20 μm. *n* = 6. ∗:*p* < 0.05, based on one-way ANOVA with Tukey’s post-hoc test.

IHC staining demonstrated that, relative to the control group, the BO group had significantly higher immunohistochemical positive area percentages for SGK1 (Figure 4G), COL1 (Figure 4H), and FN1 (Figure 4I), alongside a significantly lower positive area percentage for E-cadherin (Figure 4J) (all *p* < 0.05). Compared with the BO group, the inhibitor group displayed significantly reduced positive area percentages for SGK1, COL1, and FN1, with partial restoration of E-cadherin expression. These findings confirm the negative regulatory effect of the SGK1 inhibitor on the key ECM molecules COL1 and FN1, and suggest its potential protective role via inhibition of the EMT process. qPCR results also showed that the BO group had significantly higher mRNA expression levels of COL1 and FN1 than the control group, while the inhibitor group exhibited significantly lower mRNA expression levels of SGK1, COL1, and FN1 compared with the BO group (all *p* < 0.05) (Figures 4K–4M).

It can be found that the phenotypic improvement achieved by post-modeling SGK1 inhibitor administration was largely consistent with that of the pre-modeling administration regimen.

### SGK1 regulates BO fibrosis through the TGF-β1/SGK1 signaling pathway

We further performed RNA sequencing on HSAECs. In the SGK1 group, HSAECs were treated with EMD638683. Compared with the DMSO control group and TGF-β1-stimulated group, EMD638683 significantly downregulated the expression levels of fibrotic genes (including SGK1, COL1, and FN1) that were upregulated by TGF-β1 stimulation (Figure 5A). There were 14834 genes with no difference between the TGF-β1 group and DMSO group, and 1654 genes with difference, including 763 up-regulated genes and 891 down-regulated genes, among which SGK1 expression was found to be up-regulated (Figure 5B). There were 16172 genes with no difference between the TGF-β1+SGK1 group and the TGF-β1 group, and 161 genes with differential expression, including 94 up-regulated genes and 67 down-regulated genes, and no up-regulated SGK1 expression was observed (Figure 5C). The PPI network diagram between DMSO group and TGF-β1 group (Figure 5D) shows the interaction between SGK1 and TGF-β1, COL1 and FN1. The figure shows that TGF-β1 may affect the expression and activity of SGK1 by activating the TGF-β/SGK1 signaling pathway. It further regulates the synthesis and function of COL1 and FN1. KEGG Pathway enrichment analysis showed that compared with the DMSO group, the differentially expressed genes in the TGF-β1 group were significantly enriched in the TGF-β signaling pathway, ECM-receptor interaction, and PI3K-Akt signaling pathway (Figure 5E). Compared with the TGF-β1 group, the enrichment degree of the above pathways in the TGF-β1+SGK1 group was significantly reduced (Figure 5F). GO Cellular component enrichment analysis showed that compared with the DMSO group, the differentially expressed genes in the TGF-β1 group were significantly enriched in extracellular matrix (including collagen) related cellular components (Figure 5G). Compared with the TGF-β1 group, the enrichment degree of the differentially expressed genes in the above-mentioned cell components in the TGF-β1+SGK1 group was significantly reduced (Figure 5H).

**Figure 5:**
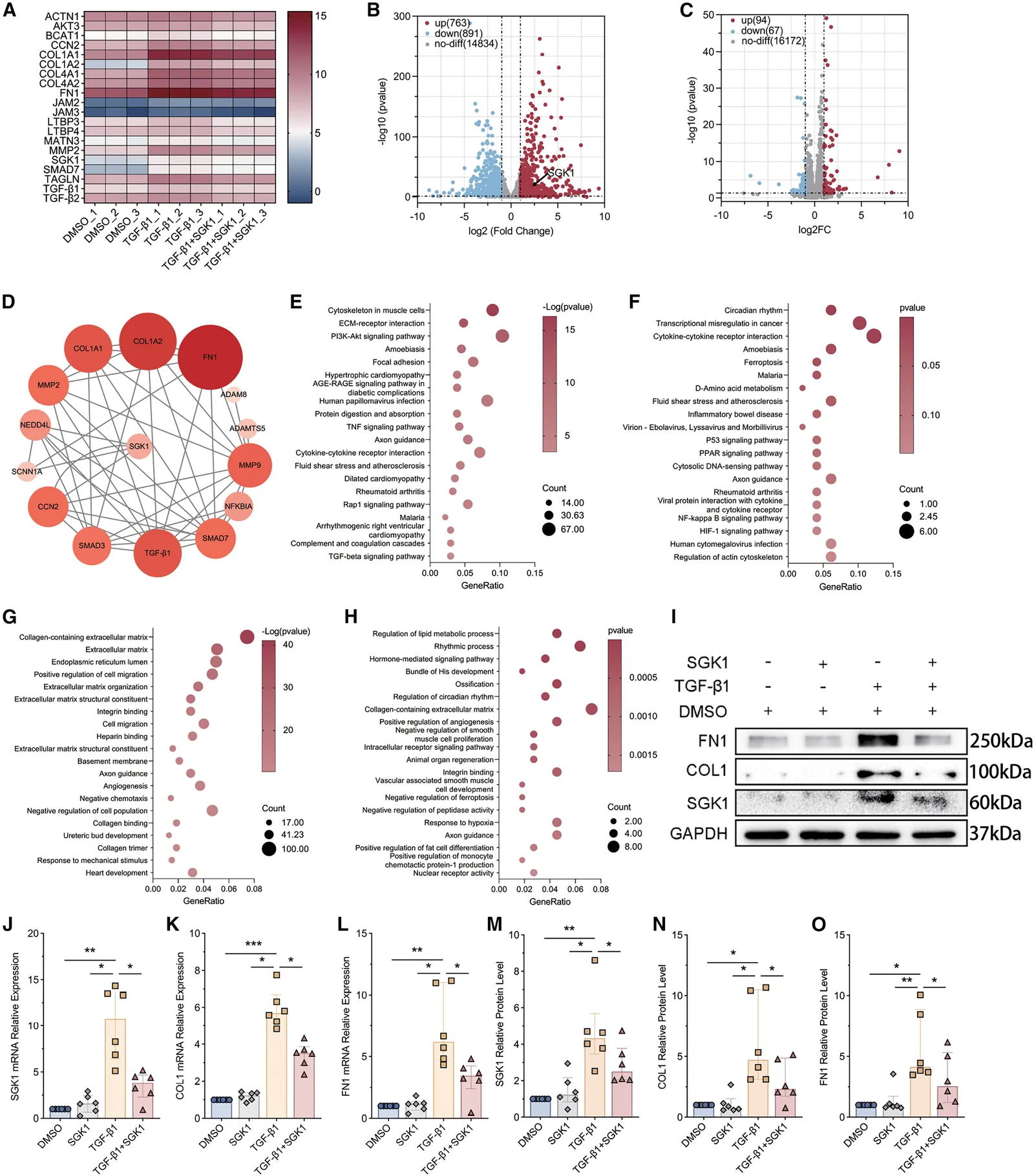
SGK1 regulates BO fibrosis through the TGF-β1/SGK1 signaling pathway HSAECs cells were divided into four groups. In the DMSO group, the solvent was added; in the TGF-β1 group, TGF-β1 was added; in the SGK1 group, SGK1 inhibitor EMD638683 was added; and in the TGF-β1 + SGK1 group, both TGF-β1 and the EMD638683 were added. (A) Heatmap of RNA-seq among the DMSO group, TGF-β1 group and TGF-β1+SGK1 group. (B) Volcano plot (TGF-β1 vs. DMSO). (C) Volcano plot (TGFβ1+SGK1 vs. TGF-β1). (D) PPI network of SGK1-related proteins (TGF-β1 vs. DMSO). (E) KEGG pathway enrichment bubble map (TGF-β1 vs. DMSO). (F) KEGG pathway enrichment bubble map (TGFβ1+SGK1 vs. TGF-β1). (G) GO cellular component enrichment bubble plot (TGF-β1 vs. DMSO). (H) GO cellular component enrichment bubble plot (TGF-β1+SGK1 vs. TGF-β1). (I) WB trend plots. (J–L) SGK1 (J), COL1 (K) and FN1 (L) mRNA relative expression levels among the DMSO group, SGK1 group, TGF-β1 group and TGF-β1+SGK1 group. (M–O) The relative protein expression levels of SGK1 (M), COL1 (N), and FN1 (O) among the DMSO group, SGK1 group, TGF-β1 group, and TGF-β1+SGK1 group. Volcano plot: Differential genes require log2 |Fold Change|>1 and *p* < 0.05. PPI network diagram: The lines between nodes represent the interactions between proteins, and the larger and darker the circles, the more interaction partners the protein has. KEGG Pathway enrichment bubble map and GO Cellular component enrichment bubble map: the most significantly enriched 20 signaling pathways and cellular components were shown, respectively. Functions with *p* < 0.05 were considered significantly enriched. ∗:*p* < 0.05; ∗∗:*p* < 0.01; and ∗∗∗:*p* < 0.001.

The results of PCR and WB showed that the mRNA and protein expression levels of SGK1, COL1, and FN1 genes in the TGF-β1 group were higher than those in the DMSO and SGK1 groups, and the expression levels of SGK1, COL1, and FN1 genes in the TGF-β1+SGK1 group were lower than those in the TGF-β1 group and the differences were statistically significant (Figures 5I–5O).

### SGK1 regulates the EMT of HSAECs through the TGF-β1/SGK1 signaling pathway

To verify the regulatory role of the TGF-β1/SGK1 signaling pathway in EMT, qPCR and western blotting were employed. HSAECs were transfected with SGK1-specific small interfering RNA (siSGK1) to knockdown SGK1 expression. For rescue experiments, HSAECs were co-transfected with siSGK1 and either the SGK1-overexpressing plasmid pcDNA3.4-SGK1 (OE or the empty vector pcDNA3.4 (NC).

qPCR and WB revealed that mRNA and protein expression levels of FN1, COL1, and SGK1 were significantly higher in the TGF-β1 group than in the DMSO group, but significantly lower in the TGF-β1+siSGK1 group compared to the TGF-β1 group, with statistically significant differences (*p* < 0.05) (Figures 6A–6C and 6F–6I). These results were consistent with those observed using the SGK1 inhibitor EMD638683 in mice. Following co-transfection, FN1 and COL1 mRNA and protein expression in the TGF-β1+siSGK1+OE group was significantly higher than in the TGF-β1+siSGK1+NC group (*p* < 0.05), preliminarily confirming that SGK1 regulates ECM secretion in HSAECs.

**Figure 6:**
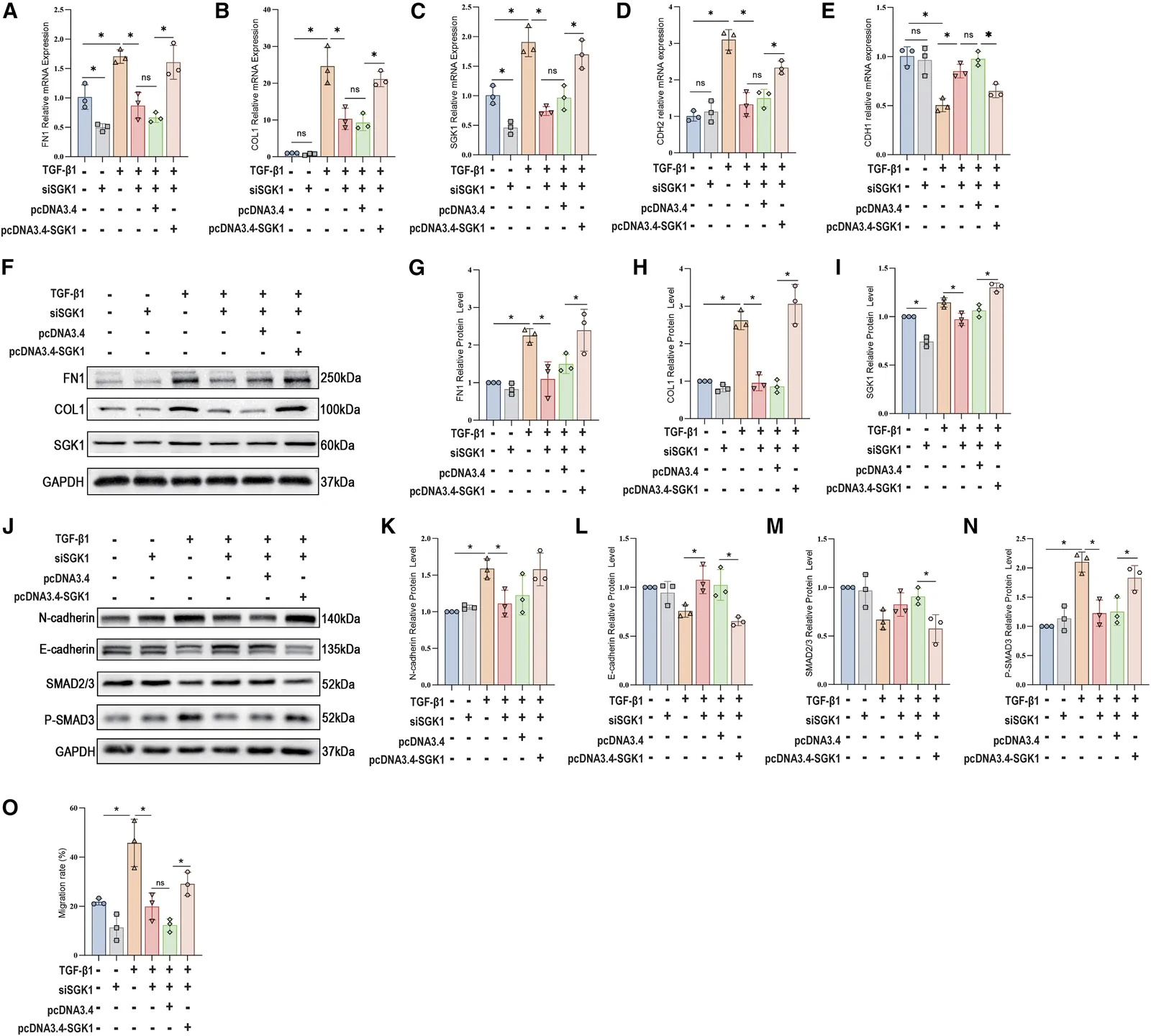
SGK1 regulates the EMT of HSAECs through the TGF-β1/SGK1 signaling pathway (A–E) FN1 (A), COL1 (B), SGK1 (C), CDH1 (D), and CDH2 (E) mRNA relative expression levels among the six groups. (F) Representative western blot bands of FN1, COL1, and SGK1 protein expression. (G–I) FN1 (G), COL1 (H), and SGK1 (I) protein relative expression levels among the six groups. (J) Representative Western blot bands of N-cadherin, E-cadherin, SMAD2/3, and P-SMAD3 protein expression. (K–N) N-cadherin (K), E-cadherin (L), SMAD2/3 (M), and P-SMAD3 (N) protein relative expression levels among the six groups. *n* = 3, ∗:*p < 0.05*, based on one-way ANOVA with Tukey’s post-hoc test.

Regarding epithelial and mesenchymal markers, N-cadherin mRNA and protein expression was significantly higher in the TGF-β1 group than in the DMSO group, but significantly lower in the TGF-β1+siSGK1 group compared to the TGF-β1 group. In contrast, E-cadherin mRNA and protein expression exhibited the opposite trend, consistent with the molecular characteristics of EMT. After co-transfection, E-cadherin mRNA and protein levels were significantly lower in the TGF-β1+siSGK1+OE group than in the TGF-β1+siSGK1+NC group, whereas N-cadherin levels showed the reverse pattern (*p* < 0.05) (Figures 6D, 6E, and 6J–6L). SMAD2/3 serves as a key marker for the activation of the TGF-β1 signaling pathway. WB analysis revealed that overexpression of SGK1 significantly reduced total SMAD2/3 protein levels while markedly increasing the expression of phosphorylated SMAD3 (*p*-SMAD3), suggesting that SGK1 may regulate the TGF-β signaling pathway by promoting SMAD3 phosphorylation (Figures 6M and 6N). These findings suggest that SGK1 may promote TGF-β1-induced EMT in HSAECs. Additionally, matrix metallopeptidase 9 (MMP9) mRNA expression was significantly higher in the TGF-β1 group than in the DMSO group, but significantly lower in the TGF-β1+siSGK1 group compared to the TGF-β1 group (*p* < 0.05), indicating enhanced invasiveness of HSAECs upon TGF-β1 stimulation. Following co-transfection, MMP9 mRNA levels in the TGF-β1+siSGK1+OE group were significantly higher than in the TGF-β1+siSGK1+NC group (*p* < 0.05) (Figure 6O), further supporting the regulatory role of SGK1 in TGF-β1-induced EMT in HSAECs.

## Discussion

The results of HE and IHC staining further confirmed the typical findings of BO, namely progressive fibrosis and obstruction of bronchioles. Experimental results showed that the BO mouse model on day 28 was more stable and successful than that on day 7.^22^^,^^23^ Many researchers have used the day 28 mouse model to study BO.^24^ In this study, SGK1 upregulation accompanied extracellular matrix accumulation and fibro-obliteration in tracheal grafts at day 28. Significant SGK1 upregulation was not detected at day 7. This time-dependent pathological progression pattern may suggest that the process of BO fibrosis can be divided into two stages: acute inflammatory stage and chronic fiber remodeling stage. The pathological characteristics of the fiber remodeling stage are more consistent with the chronic progression of BO after lung transplantation, suggesting that SGK1 may play a key role in the middle and late stages of BO fibrosis. Based on the results of this pathology and volcano plot, in order to obtain a more stable BO mouse model and more reliable experimental results, we only selected the model of BO mice on day 28 for further study in the follow-up study.

KEGG enrichment analysis showed that the differentially expressed genes were significantly enriched in Th1 and Th2 cell differentiation pathways, suggesting that the pathogenesis of BO is closely related to immune regulation imbalance.^25^ Th1/Th2 polarization is the core mechanism of adaptive immunity, and Th1-type responses mainly mediate antiviral immunity, while Th2-type responses are associated with allergic and fibrotic diseases.^26^ Existing literature indicates that SGK1 is a key regulatory factor in T cell differentiation and function, specifically modulating the dynamic balance among Th1, Th2, and Th17 subsets.^27^ In the context of allograft rejection, SGK1-mediated shifts toward pathogenic T-helper phenotypes may exacerbate initial alloimmune injury and subsequently promote fibrotic remodeling. This finding suggests that SGK1 may play a dual role in the pathogenesis of BO—both participating in epithelial-mesenchymal transition and regulating adaptive immune responses. In addition, the enrichment of graft rejection pathways may reflect the immune rejection mechanism of BO in post-transplant patients, suggesting that therapeutic strategies that inhibit T cell activation may have potential efficacy. ECM deposition and remodeling are the core pathological features of airway fibrosis in BO.^28^

Reanalysis of GSE225159 identified increased SGK1 expression in TGF-β1-induced myofibroblasts compared with untreated MRC-5 lung fibroblasts. This finding extends the observed association between SGK1 and profibrotic responses to an additional human cell model. Together with the HSAEC findings, it supports investigating SGK1 across epithelial and stromal compartments involved in airway remodeling.

Numerous studies have demonstrated that EMD638683 exerts a selective inhibitory effect on SGK1.^18^^,^^20^^,^^29^^,^^30^ Compared with other SGK1 inhibitors (e.g., GSK650394), EMD638683 demonstrates greater selectivity for SGK1.^29^^,^^31^ EMD638683 could significantly inhibit the TGF-β1-induced upregulation of fibrosis-related gene expression, but there was no upregulation of SGK1 expression after EMD638683 intervention. Differential genes were enriched in ECM-receptor interaction pathways and significantly reduced ECM components, suggesting that SGK1 plays an important regulatory role in TGF-β1-mediated extracellular matrix deposition,^5^^,^^32^ and SGK1 is a key mediator of TGF-β1-induced ECM remodeling.

In conclusion, SGK1 expression was significantly up-regulated on day 28 in the BO mouse model, which was closely related to the high expression of ECM fibrosis genes such as COL1 and FN1. *In vitro* experiments further confirmed that SGK1 regulated EMT and fibrosis progression through the TGF-β1 signaling pathway. Its inhibitor EMD638683 could effectively alleviate this process, suggesting that SGK1 might be an important target for the regulation of BO-related fibrosis. These findings provided a theoretical and experimental basis for the treatment strategy of BO after lung transplantation in the clinic.

### Limitations of the study

This study has several limitations. The heterotopic tracheal transplantation model reproduces features of airway fibro-obliteration but uses large-airway grafts outside the native pulmonary environment that do not participate in normal ventilation.^33^^,^^34^ Its findings therefore require validation in models that more closely reflect small-airway disease after lung transplantation. Only male mice were studied, precluding assessment of sex-dependent responses. Moreover, the epithelial experiments used a single immortalized HSAEC line and did not capture donor variability or interactions with immune and stromal cells. Direct validation in patient-derived samples is also lacking; the reanalyzed public dataset represents a fibroblast-to-myofibroblast transition model rather than a clinical BO cohort.

Although SGK1 knockdown and re-expression experiments support its involvement in the cellular responses, systemic EMD638683 treatment does not establish which cell types mediate the effects *in vivo* or exclude off-target contributions. Cell-type-specific genetic studies would help resolve these questions. The contribution of epithelial cells to collagen-producing myofibroblasts *in vivo* was not directly determined. Similarly, the observed changes in SMAD3 phosphorylation do not establish direct phosphorylation of SMAD3 by SGK1. Finally, treatment initiated on postoperative day 7 reduced subsequent pathological changes, but this design does not establish reversal of mature fibrosis. Longer-term studies are needed to determine the durability and safety of treatment.

## Resource availability

### Lead contact

Requests for further information and resources should be directed to and will be fulfilled by the lead contact, Dehui Chen (dehuichen2022@163.com).

### Materials availability

Plasmid sequences and maps are available from the lead contact upon reasonable request.

### Data and code availability

• The raw RNA sequencing data reported in this paper have been deposited in the Gene Expression Omnibus (https://www.ncbi.nlm.nih.gov/geo/query/acc.cgi?acc=GSE334327 and https://www.ncbi.nlm.nih.gov/geo/query/acc.cgi?acc=GSE349082), and the datasets are already publicly available.
• This paper does not report original code.
• Any additional information required to reanalyze the data reported in this paper is available from the lead contact upon request.

## Acknowledgments

This work was supported by the Guangzhou Science and Technology Program Project (grant no 2024A03J1203), the 10.13039/501100021171Basic and Applied Basic Research Foundation of Guangdong Province (grant no 2021A1515220043), and the Guangzhou Science and Technology Program Project (grant no 2024A04J3538).

## Author contributions

L.Z. and H.W.L. performed multiple experiments, data acquisition, and data analysis. S.H.X. and J.X.L. performed bioinformatics analysis and data processing. H.W.L., L.Z., and Y.H.W. collected experimental data and participated in revising the manuscript. S.X.T., C.Y.L., and X.W.C. collected and sorted cell experimental data. D.H.C. contributed to the experimental design and participated in completing the relevant experiments.

## Declaration of interests

The authors declare no competing interests.

## STAR★Methods

### Key resources table

**Table:** 

REAGENT or RESOURCE	SOURCE	IDENTIFIER
**Antibodies**		

Rabbit Anti-SGK1 antibody. IHC, 1:200	Boster	Cat# BM4452
Mouse monoclonal anti-Collagen Type I. IHC, 1:500	Proteintech	Cat# 66761-1-Ig
Rabbit monoclonal anti-Fibronectin. IHC, 1:300	Boster	Cat# M00564-3
Rabbit polyclonal anti-E-cadherin. IHC, 1:400; WB, 1:1000	Affinity Biosciences	Cat# AF0131
Rabbit polyclonal anti-SGK1. WB, 1:1000	Affinity Biosciences	Cat# AF6001
Rabbit polyclonal anti-Collagen I. WB, 1:2000	Servicebio	Cat# GB11022-100
Rabbit polyclonal anti-Fibronectin. WB, 1:1500	Servicebio	Cat# GB114491-100
Rabbit Anti-N-cadherin antibody. WB, 1:1000	Servicebio	Cat# GB111273-100
Rabbit polyclonal anti-phospho-Smad3 (Ser425). WB, 1:1000	Affinity Biosciences	Cat# AF3362
Rabbit polyclonal anti-Smad2/3. WB, 1:1000	Affinity Biosciences	Cat# AF6367
Rabbit monoclonal anti-GAPDH. WB, 1:4000	Cell Signaling Technology	Cat# 5174T
Goat anti-mouse IgG (HRP). IHC, 1:200	Servicebio	Cat# GB23301
Goat anti-rabbit IgG H&L (HRP). IHC, 1:200	Servicebio	Cat# GB23303
Goat anti-rabbit IgG H&L (HRP). WB, 1:3000	Abcam	Cat# ab6721

**Chemicals, peptides, and recombinant proteins**		

EMD638683 (SGK1 inhibitor)	MedChemExpress (MCE)	Cat# HY-15193
Recombinant human TGF-β1	Proteintech	Cat# HZ-1011
Lipofectamine 3000 Transfection Reagent (including P3000 reagent)	Invitrogen/Thermo Fisher Scientific	Cat# L3000008
Fetal bovine serum (FBS)	Vazyme	Cat# F101-03
Dimethyl sulfoxide (DMSO)	Biosharp	Cat# BL165B
Polyethylene glycol 300 (PEG300)	MedChemExpress (MCE)	Cat# HY-Y0873
Tween 80	Selleck	Cat# S6702
Citrate antigen retrieval solution (20×, pH 6.0)	Servicebio	Cat# G1202
Tris-EDTA antigen retrieval solution (20×, pH 9.0)	Servicebio	Cat# G1203
Tris-EDTA antigen retrieval solution (20×, pH 8.0)	Servicebio	Cat# G1206
Tribromoethanol solution (1.25%, ready-to-use; Avertin)	Leagene	Cat# R00655
RNAfollow tissue RNA preservation solution	NCM	Cat# M6100
Paraformaldehyde buffer (4%)	Biosharp	Cat# BL539A
DMEM/F12 medium	Gibco/Thermo Fisher Scientific	Cat# C11330500BT
Opti-MEM medium	Gibco/Thermo Fisher Scientific	Cat# 51985034
Penicillin–streptomycin	Biosharp	Cat# BL141A
RIPA lysis buffer	Beyotime	Cat# P0013B
Phenylmethylsulfonyl fluoride (PMSF)	Beyotime	Cat# ST505
SDS-PAGE loading buffer (5×)	Servicebio	Cat# G2075-10 ML
Skim milk	Servicebio	Cat# GC310001
Bovine serum albumin (BSA)	Servicebio	Cat# GC305010
TBST buffer	Servicebio	Cat# G2150-1L

**Critical commercial assays**		

Fast Cell RNA Extraction Kit	GOONIE	Cat# 400–100
Cell/Tissue RNA Extraction Kit (for tissue samples)	GOONIE	Cat# 400–105
HiScript II Q RT SuperMix for qPCR (+gDNA wiper)	Vazyme	Cat# R223-01
ChamQ Universal SYBR qPCR Master Mix	Vazyme	Cat# Q711-02
Omni-Easy One-Step PAGE Gel Fast Preparation Kit (10%)	Epizyme	Cat# PG212
Enhanced chemiluminescence (ECL) detection reagent	NCM	Cat# P10300
Hematoxylin-eosin (H&E) HD constant dye kit	Servicebio	Cat# G1076
Masson trichrome staining kit	Servicebio	Cat# G1006
DAB Chromogenic Kit	Servicebio	Cat# G1212

**Deposited data**		

RNA-seq data from mouse tracheal samples	This paper	GEO: GSE334327
RNA-seq data from HSAEC experiments	This paper	GEO: GSE349082
Public RNA-seq data from MRC-5 lung fibroblasts and derived myofibroblasts	Xu et al.^21^; NCBI GEO	GEO: GSE225159 Control (FB): GSM7040245, GSM7040246, GSM7040247 TGF-β1-induced MFB: GSM7040248, GSM7040249, GSM7040250

**Experimental models: Cell lines**		

Human small airway epithelial cell line (HSAEC)	ginbio (Guangzhou, China)	Cat# GB20273

**Experimental models: Organisms/strains**		

Mouse: C57BL-6J	Guangzhou Ruige Biological Technology Co., Ltd.	Cat# C57-6J002
Mouse: BALB/C	Guangzhou Ruige Biological Technology Co., Ltd.	Cat# B/C-002

**Oligonucleotides**		

Mouse GAPDH primers Forward: TGTGTCCGTCGTGGATCTGA Reverse: TTGCTGTTGAAGTCGCAGGAG	IGEbio (Guangzhou, China)	IGEbio (Guangzhou, China)
Mouse FN1 primers Forward: ACAATGCCGTGGTCCTAACA Reverse: AGTGAATGAGTTGGCGGTGA	IGEbio (Guangzhou, China)	IGEbio (Guangzhou, China)
Mouse COL1 primers Forward: GACATGTTCAGCTTTGTGGACCTC Reverse: GGGACCCTTAGGCCATTGTGTA	IGEbio (Guangzhou, China)	IGEbio (Guangzhou, China)
Mouse SGK1 primers Forward: CTGCTCGAAGCACCCTTACC Reverse: TCCTGAGGATGGGACATTTTCA	IGEbio (Guangzhou, China)	IGEbio (Guangzhou, China)
Human GAPDH primers Forward: GCACCGTCAAGGCTGAGAAC Reverse: TGGTGAAGACGCCAGTGGA	IGEbio (Guangzhou, China)	IGEbio (Guangzhou, China)
Human FN1 primers Forward: GCAGGCTCAGCAAATGGTTC Reverse: GTCCGCTCCCACTGTTGATTTA	IGEbio (Guangzhou, China)	IGEbio (Guangzhou, China)
Human COL1 primers Forward: CTGCAACATGGAGACTGGTG Reverse: CGCCATACTCGAACTGGAATC	IGEbio (Guangzhou, China)	IGEbio (Guangzhou, China)
Human SGK1 primers Forward: ATAGCTAGCACCCTGTTAGGGCTTG Reverse: ATATCTCGAGCAACGGCTCTGACTG	IGEbio (Guangzhou, China)	IGEbio (Guangzhou, China)
Human CDH1 primers Forward: ATGGCTGAAGGTGACAGAGC Reverse: TGCATTCCCGTTGGATGACA	IGEbio (Guangzhou, China)	IGEbio (Guangzhou, China)
Human CDH2 primers Forward: AGGCTTCTGGTGAAATCGCA Reverse: AGAGGCTGTCCTTCATGCAC	IGEbio (Guangzhou, China)	IGEbio (Guangzhou, China)
SGK1-targeting siRNA (siSGK1) Sense: GGAAUGUUCUGUUGAAGAAdTdT Antisense: UUCUUCAACAGAACAUUCCdTdT	IGEbio (Guangzhou, China)	IGEbio (Guangzhou, China)
Non-targeting control siRNA Sense: UUCUCCGAACGUGUCACGUdTdT Antisense: ACGUGACACGUUCGGAGAAdTdT	IGEbio (Guangzhou, China)	IGEbio (Guangzhou, China)

**Recombinant DNA**		

pcDNA3.4-CMV-Homo-SGK1-EF1a-CopGFP-neo	IGEbio (Guangzhou, China)	Construct ID: IGEB8349-1 Plasmid length: 9,184 bp
pcDNA3.4-CMV-MCS-EF1a-CopGFP-neo	IGEbio (Guangzhou, China)	Construct ID: IGEB8349 Plasmid length: 7,615 bp

**Software and algorithms**		

IBM SPSS Statistics 27.0	IBM	Version 27.0
GraphPad Prism	GraphPad Software	Version 10.1.2
Fiji (ImageJ)	Fiji project	ImageJ version 1.54P https://github.com/fiji
SOAPnuke	BGI	Version 2.3 https://github.com/BGI-flexlab/SOAPnuke
HISAT2	HISAT2 developers	Version 2.0.4 https://daehwankimlab.github.io/hisat2/
RSEM	Dewey Lab	Version 1.2.8 https://github.com/deweylab/RSEM
Cytoscape	Cytoscape developers	Version 3.10.4
Bowtie2	Bowtie2 developers	Version 2.2.5 https://bowtie-bio.sourceforge.net/bowtie2/index.shtml
DESeq2	Bioconductor	Version 1.42.0
Metascape	Metascape team	Version 3.5.20260701 https://metascape.org/gp/

### Experimental model and study participant details

#### Mice

Male C57BL-6J mice (Cat# C57-6J002) and BALB/C mice (Cat# B/C-002), aged 6–8 weeks and weighing approximately 20 g, were obtained from Guangzhou Ruige Biological Technology Co., Ltd. (Guangzhou, China). Mice were acclimatized for 7 days and maintained under specific pathogen-free conditions with *ad libitum* access to food and water. Animals were housed at no more than five mice per cage under a 12-h light/12-h dark cycle, with a light intensity of 15–20 lux, a temperature of 20°C–22°C with a daily fluctuation of no more than 4°C, and a relative humidity of 50–60%.

Male mice were used to reduce the influence of estrous-cycle variation on the experimental outcomes.

#### Human small airway epithelial cells

The immortalized human small airway epithelial cell line HSAEC (ginbio, Guangzhou, China; Cat# GB20273) was cultured in DMEM/F12 medium (Gibco, C11330500BT) supplemented with 10% fetal bovine serum (Vazyme, F101-03), 100 U/mL penicillin, and 0.1 mg/mL streptomycin (Biosharp, BL141A), at 37°C in a humidified atmosphere containing 5% CO_2_. Cells at passages 3–5 were used for experiments. The HSAEC cell line was not independently authenticated in this study, and the cultures were not tested for mycoplasma contamination.

#### Ethics review

Animal care and experiment procedures were conducted in accordance with the “Guiding Principles in the Care and Use of Animals” (China) and approved by the Animal Care and Use Committee of Guangzhou Medical University (Approval No. GZMU202300X).

### Method details

#### Heterotopic tracheal transplantation

A heterotopic tracheal transplantation model was established as previously described by Hertz et al.^35^ C57BL-6J mice served as recipients. Tracheas from C57BL-6J donors were used for syngeneic controls, whereas tracheas from BALB/C donors were used to establish the allogeneic BO model. Mice were assigned to experimental groups using computer-generated randomization. Anesthesia was induced by intraperitoneal injection of ready-to-use 1.25% tribromoethanol solution (Avertin; Leagene, R00655) at 0.2 mL per 10 g body weight. All surgical instruments were disinfected before use. No analgesics were administered during or after surgery.

Following anesthesia, hair around the donor surgical site was removed, and the skin was disinfected three times with povidone-iodine. A midline cervical incision was made from the level of the larynx to the sternum. Subcutaneous tissues were dissected laterally to expose the trachea. The entire trachea, extending from the larynx to the tracheal bifurcation, was excised, rinsed in saline at 4°C for 10 s, and temporarily held in saline at 4°C until implantation.

In each recipient, a midline incision no longer than 1 cm was made in the posterior cervical region, and the subcutaneous tissue was carefully separated to create a small pocket. Bleeding was minimized during dissection; minor bleeding was gently cleared with saline-moistened gauze. One tracheal graft was placed into the pocket per recipient. The tissues surrounding the pocket and the skin were closed in layers using surgical sutures, and the skin incision was disinfected again. The total surgical time was less than 15 min.

After surgery, mice were placed on a heating pad maintained at 37°C until they had fully recovered from anesthesia, as indicated by the return of the righting reflex and spontaneous movement. Mice were observed at least twice daily during the first 72 h and daily thereafter until the end of the experiment. Monitoring included body weight, general condition, mobility, and signs of infection or dehiscence at the incision. Sutures were removed 7–10 days after surgery, or at euthanasia if this occurred earlier. Prespecified humane endpoints were a loss of more than 20% of baseline body weight; persistent hunched posture, lack of grooming, or impaired mobility; or inability to access food or water. Animals showing persistent signs of severe distress were euthanized immediately.

For endpoint sampling, recipients were anesthetized, the posterior cervical region was shaved, and the surgical site was disinfected with povidone-iodine. The skin and underlying tissues were opened in layers, and the graft was carefully separated from the surrounding tissue and removed. Each graft was rinsed in saline at 4°C for 10 s before allocation to the RNA preservation or histology procedures described below. All euthanasia was performed by cervical dislocation under anesthesia. Grafts were collected on postoperative days 7, 14, and 28 for evaluation of pathological progression.

#### EMD638683 treatment in mice

An EMD638683 stock solution was prepared by dissolving 50 mg EMD638683 powder (MCE, HY-15193) in 1 mL DMSO (Biosharp, BL165B) and mixing thoroughly by repeated pipetting to obtain a concentration of 50 mg/mL (0.05 mg/μL). The stock was stored at −20°C protected from light. The working solution was freshly prepared by sequentially combining 10% of the DMSO stock solution, 40% PEG300 (MCE, HY-Y0873), 5% Tween 80 (Selleck, S6702), and 45% saline, giving a final EMD638683 concentration of 5 mg/mL. Mice received the working solution once daily by oral gavage at 0.004 mL/g body weight, equivalent to 20 mg/kg/day. In the prophylactic experiment, dosing began 2 days before transplantation; in the therapeutic experiment, dosing began on postoperative day 7. Each experiment included syngeneic vehicle controls, allogeneic vehicle controls, and allogeneic EMD638683-treated mice. Vehicle controls received the corresponding drug-free solvent formulation on the same schedule. Body weight and health status were monitored throughout the experiments.

#### Cell stimulation and inhibitor treatment

When HSAECs reached approximately 70% confluence, the medium was replaced with fresh complete culture medium. According to the experimental group, cells were treated with recombinant human TGF-β1 (Proteintech, HZ-1011; 10 ng/mL), EMD638683 (MCE, HY-15193; approximately 3 μM), or both. TGF-β1 and EMD638683 were added simultaneously in the combined-treatment group. The pharmacological experiments comprised DMSO vehicle, EMD638683 alone, TGF-β1 alone, and TGF-β1 plus EMD638683 groups. All treatments lasted 48 h, after which cells were harvested for RNA extraction, protein extraction, or RNA sequencing, as appropriate.

For the EMD638683-only group, a stock solution was prepared by dissolving 50 mg EMD638683 (MCE, HY-15193) in 1 mL DMSO (Biosharp, BL165B), with repeated pipetting to mix thoroughly, yielding 50 mg/mL (50 μg/μL). The stock was stored at −20°C protected from light. An intermediate dilution was prepared by adding 1 μL stock to 45.7 μL complete medium in a 1.5-mL microcentrifuge tube. After mixing, 2 μL of this dilution was added to each well of a six-well plate containing 2 mL complete medium, yielding approximately 3 μM EMD638683.

For the TGF-β1-only group, TGF-β1 (Proteintech, HZ-1011) was dissolved in DMSO at a stock concentration of 0.467 μg/μL. The stock was stored at −80°C. One microliter of stock was mixed with 45.7 μL complete medium, and 2 μL of this dilution was added to each well containing 2 mL complete medium, yielding approximately 10 ng/mL TGF-β1.

For the combined-treatment group, EMD638683 and TGF-β1 were co-dissolved in DMSO to prepare a mixed stock containing 50 μg/μL EMD638683 and 0.467 μg/μL TGF-β1. The mixed stock was stored at −80°C. One microliter of this mixed stock was diluted with 45.7 μL complete medium, and 2 μL of the dilution was added to each well containing 2 mL complete medium. The resulting concentrations were approximately 3 μM EMD638683 and 10 ng/mL TGF-β1.

For the DMSO vehicle group, 1 μL DMSO was mixed with 45.7 μL complete medium, and 2 μL of this mixture was added to each well containing 2 mL complete medium. The same dilution and addition volumes were used for all four groups, giving a matched final DMSO concentration of approximately 0.00214% (v/v).

#### SGK1 knockdown and rescue

HSAECs were transfected with SGK1-targeting siRNA or a non-targeting control siRNA using Lipofectamine 3000 (Invitrogen, L3000008). The siSGK1 sense and antisense sequences were 5′-GGAAUGUUCUGUUGAAGAAdTdT-3′ and 5′-UUCUUCAACAGAACAUUCCdTdT-3′, respectively. The control siRNA sense and antisense sequences were 5′-UUCUCCGAACGUGUCACGUdTdT-3′ and 5′-ACGUGACACGUUCGGAGAAdTdT-3′, respectively. Both siRNAs were synthesized by IGEbio (Guangzhou, China).

siRNA stocks were prepared by dissolving 2.5 nmol siRNA in 125 μL nuclease-free water to obtain a concentration of 20 μM. The siRNA was allowed to dissolve for 30 min at room temperature, with intermittent vortexing, and the stocks were stored at −80°C.

Transfection was performed when HSAECs in six-well plates reached approximately 70% confluence. The culture medium was removed, cells were washed twice with PBS, and 1.5 mL Opti-MEM medium (Gibco, 51985034) was added to each well. For each well, 5 μL of the 20 μM siRNA stock was diluted in 250 μL Opti-MEM. In a separate tube, 5 μL Lipofectamine 3000 was diluted in 250 μL Opti-MEM. After incubation for 5 min at room temperature, the two dilutions were combined, gently mixed by pipetting three to five times, and incubated for a further 20 min at room temperature. A 500-μL aliquot of the transfection mixture was added to each well, yielding an siRNA concentration of approximately 50 nM. The medium was replaced with complete culture medium 6 h after addition of the transfection complexes. For the siRNA plus TGF-β1 groups, TGF-β1 was added at this medium change at a final concentration of 10 ng/mL and maintained for 42 h. Cells were harvested for RNA or protein extraction 48 h after addition of the transfection complexes.

For SGK1 re-expression experiments, cells were co-transfected with siSGK1 and either pcDNA3.4-CMV-Homo-SGK1-EF1a-CopGFP-neo (IGEbio; construct IGEB8349-1; 9,184 bp) or the corresponding empty vector pcDNA3.4-CMV-EF1a-CopGFP-neo. siRNA and primer sequences and construct identifiers are listed in the key resources table.

For co-transfection, HSAECs were grown in six-well plates to approximately 70% confluence. The medium was removed, cells were washed twice with PBS, and 1.5 mL Opti-MEM (Gibco, 51985034) was added to each well. For each well, 5 μL of the 20 μM siRNA stock, 3 μg plasmid DNA (approximately 3.21 μL of the 934 ng/μL SGK1 expression plasmid stock or 2.86 μL of the 1048 ng/μL empty-vector stock), and 5 μL P3000 reagent were added to 250 μL Opti-MEM. In a separate tube, 5 μL Lipofectamine 3000 (Invitrogen, L3000008) was added to 250 μL Opti-MEM. After incubation for 5 min at room temperature, the diluted transfection reagent was combined with the plasmid–siRNA mixture, gently mixed by pipetting three to five times, and incubated for 20 min at room temperature. A 500-μL aliquot of the resulting complexes was added to each well. The medium was replaced with complete culture medium 6 h after addition of the complexes. For co-transfection groups requiring TGF-β1 stimulation, TGF-β1 was added at this medium change at 10 ng/mL and maintained for 42 h. Cells were harvested for RNA or protein extraction 48 h after addition of the complexes.

#### RNA extraction and RT-qPCR

At the experimental endpoint, harvested tracheal grafts designated for RT-qPCR and RNA sequencing were rinsed in saline at 4°C for 10 s and preserved in RNAfollow tissue RNA preservation solution (NCM, M6100) at −20°C. RNA was extracted within 3 days of tissue collection. Total RNA was isolated from HSAECs using the cell RNA extraction kit (GOONIE, 400–100) and from tracheal tissues using the tissue RNA extraction kit (GOONIE, 400–105). RNA concentration and absorbance ratios were measured using a NanoDrop 2000 spectrophotometer (Thermo Scientific, ND-2000). RNA acceptance criteria were a concentration of at least 100 ng/μL, an A260/A280 ratio of 1.9–2.1, and an A260/A230 ratio of at least 1.8.

Reverse transcription was performed using HiScript II Q RT SuperMix for qPCR (+gDNA wiper) (Vazyme, R223-01) according to the manufacturer’s protocol (version 22.1), with a final reaction volume of 20 μL. For genomic DNA removal, approximately 200 ng total RNA, 4 μL 4× gDNA wiper Mix, and RNase-free water were combined to a total volume of 16 μL and incubated at 42°C for 2 min. Subsequently, 4 μL 5× HiScript II qRT SuperMix II was added, and reverse transcription was performed at 50°C for 15 min, followed by 85°C for 5 s.

qPCR was performed using ChamQ Universal SYBR qPCR Master Mix (Vazyme, Q711-02) on a CFX96 Touch Deep Well Real-Time PCR Detection System (Bio-Rad, 3600037). The manufacturer’s reaction setup (version 23.1) was scaled proportionally to a final volume of 10 μL, containing 5 μL 2× Master Mix, 0.2 μL each of the 10 μM forward and reverse primers (0.2 μM final concentration each), 1 μL undiluted cDNA, and 3.6 μL water. The amplification protocol comprised initial denaturation at 95°C for 30 s, followed by 40 cycles of 95°C for 10 s and 60°C for 30 s, with fluorescence acquisition during the 60°C step. Melt-curve analysis was performed using the instrument’s default acquisition program.

Relative expression was calculated using the 2ˆ−ΔΔCt method. Primers were synthesized by IGEbio and are listed in the key resources table.

#### Histology and immunohistochemistry

At the experimental endpoint, harvested tracheal grafts for histology were rinsed in saline at 4°C for 10 s and held in 4% paraformaldehyde buffer (Biosharp, BL539A) at 4°C before processing, paraffin embedding, and sectioning at 3–5 μm. Hematoxylin and eosin (H&E) staining was performed using the Servicebio G1076 kit. Sections were deparaffinized in two changes of clearing solution for 20 min each, followed by two changes of absolute ethanol and 75% ethanol for 5 min each, and rinsed in water. After pretreatment for 1 min, sections were stained with hematoxylin for 3–5 min, differentiated, blued, exposed to 95% ethanol for 1 min, and stained with eosin for 15 s. Sections were dehydrated, cleared, and mounted.

Masson’s trichrome staining was performed using the Servicebio G1006 kit. Deparaffinized and rehydrated sections were incubated in solution A overnight, stained with a 1:1 mixture of solutions B and C for 1 min, briefly differentiated, and rinsed. Sections were then incubated in solution D for 6 min and solution E for 1 min, followed directly by solution F for 2–30 s without an intervening wash. Sections were differentiated with 1% acetic acid, dehydrated, cleared, and mounted. Collagen was identified by blue staining.

For immunohistochemistry, sections were deparaffinized in three changes of clearing solution for 10 min each and rehydrated through three changes of absolute ethanol for 5 min each, followed by water. The antigen retrieval buffer and conditions were selected according to the tissue. Retrieval reagents comprised citrate buffer, pH 6.0 (Servicebio, G1202), and Tris-EDTA buffers, pH 9.0 (G1203) or pH 8.0 (G1206), supplied as 20× concentrates. After cooling and PBS washing, endogenous peroxidase was blocked with 3% hydrogen peroxide for 25 min at room temperature in the dark. Sections were washed and blocked with 3% BSA for 30 min at room temperature.

Sections were incubated overnight at 4°C with antibodies against SGK1 (Boster, BM4452; 1:200), collagen type I (Proteintech, 66761-1-Ig; 1:500), fibronectin (Boster, M00564-3; 1:300), or E-cadherin (Affinity, AF0131; 1:400). After three PBS washes of 5 min each, sections were incubated with the appropriate HRP-conjugated goat anti-mouse IgG (Servicebio, GB23301; 1:200) or goat anti-rabbit IgG (Servicebio, GB23303; 1:200) for 50 min at room temperature. Following PBS washing, staining was developed using DAB (Servicebio, G1212), monitored microscopically, and stopped by washing with water. Sections were counterstained with hematoxylin for approximately 3 min, differentiated, blued, dehydrated, cleared, and mounted. Images were acquired using a Nikon ECLIPSE E100 microscope.

#### Histological quantification

Histological images were quantified using Fiji with ImageJ version 1.54P. Endpoints included epithelial loss, luminal occlusion, collagen content assessed by Masson’s trichrome staining, and the percentage of IHC-positive area for SGK1, collagen type I, fibronectin, and E-cadherin. Two independent investigators assessed the sections while blinded to group allocation.

#### Western blotting

Total cellular protein was extracted using ice-cold RIPA buffer (Beyotime, P0013B) supplemented with PMSF (Beyotime, ST505). Cells were lysed on ice for 20 min, and lysates were centrifuged at 12,000 rpm for 15 min at 4°C. Tracheal tissue is ground and then lysed. The supernatants were collected and mixed with 5× SDS-PAGE loading buffer (Servicebio, G2075-10 ML) to a final buffer concentration of 1×. Samples were heated in a dry block at 95°C for 10 min and stored at −80°C. Samples were loaded at 10 μL per lane onto 15-well, 10% SDS-polyacrylamide gels (Epizyme, PG212). Electrophoresis was performed at 80 V until the bands exited the stacking gel and then at 120 V. Proteins were electrotransferred to PVDF membranes (Merck Millipore, IPVH00010) at 400 mA for 90 min.

Membranes were blocked for 1 h at room temperature in TBST (Servicebio, G2150-1L) containing 5% skim milk (Servicebio, GC310001). For phosphorylated proteins, 5% BSA (Servicebio, GC305010) was used instead of skim milk. After blocking, membranes were washed once with TBST for 5 min. Primary antibodies were diluted in 5% BSA and incubated with the membranes overnight at 4°C.

Membranes were incubated with primary antibodies against SGK1 (Affinity, AF6001; 1:1,000), collagen I (Servicebio, GB11022-100; 1:2,000), fibronectin (Servicebio, GB114491-100; 1:1,500), E-cadherin (Affinity, AF0131; 1:1,000), N-cadherin (Servicebio, GB111273-100; 1:1,000), phospho-SMAD3 (Ser425; Affinity, AF3362; 1:1,000), SMAD2/3 (Affinity, AF6367; 1:1,000), and GAPDH (Cell Signaling Technology, 5174T; 1:4,000).

After primary antibody incubation, membranes were washed three times with TBST for 5, 5, and 10 min, respectively. Subsequently, membranes were incubated with HRP-conjugated goat anti-rabbit IgG (Abcam, ab6721; 1:3,000) diluted in TBST for 1 h at room temperature. Following secondary antibody incubation, membranes were washed again with TBST for 5, 5, and 10 min. Signals were developed using ECL substrate (NCM, P10300) and acquired using a Pxi 9 chemiluminescence imaging system (Syngene). Band intensities were quantified using Fiji with ImageJ version 1.54P.

### Quantification and statistical analysis

#### RNA sequencing

RNA from tracheal grafts was submitted to BGI Genomics (Shenzhen, China) for RNA sequencing. Poly(A)-selected, strand-specific libraries were prepared using the DNBSEQ mRNA-seq protocol and sequenced on the DNBSEQ-G400 platform. Paired-end 150-bp sequencing was reported in the original manuscript.

#### RNA-seq data processing

For the mouse tracheal dataset, low-quality reads and adapter sequences were removed using SOAPnuke v2.3 with parameters -l 20 -q 0.2 -n 0.05. Clean reads were aligned to the Mus musculus reference genome GRCm39 (GCF_000001635.27) using HISAT2 v2.0.4. For expression quantification, clean reads were aligned to reference gene sequences using Bowtie2 v2.2.5, and gene expression levels were calculated using RSEM v1.2.8. Differential expression analysis was performed using DESeq2 v1.42.0. The stated differential-expression cutoff was |log_2_(fold change)| > 1 and *p* < 0.05. Bioinformatics result plots were prepared using GraphPad Prism 10.1.2. KEGG enrichment analysis of differentially expressed genes was performed using Metascape v3.5.20260701. The enrichment plots displayed the 20 most significant terms. Protein–protein interaction networks were visualized using Cytoscape v3.10.4.

#### Public transcriptomic data analysis

Public RNA-seq data from GSE225159 were obtained from the Gene Expression Omnibus. The analysis included three untreated MRC-5 human lung fibroblast samples (GSM7040245, GSM7040246, and GSM7040247) and three TGF-β1-induced myofibroblast samples (GSM7040248, GSM7040249, and GSM7040250). The MSC co-culture samples were not included. This comparison represents an *in vitro* fibroblast-to-myofibroblast model. SGK1 expression and transcriptome-wide differences were assessed between the two groups, and sample distributions were visualized using UMAP.

#### Statistical analysis

Statistical analyses were performed using IBM SPSS Statistics 27.0 and GraphPad Prism 10.1.2. Normality was assessed using the Shapiro–Wilk test. Two-group comparisons used an unpaired Student’s *t* test for normally distributed data or a Mann–Whitney U test for non-normally distributed data. Multiple-group comparisons used one-way ANOVA followed by Tukey’s test for normally distributed data or the Kruskal–Wallis test followed by Dunn’s test for non-normally distributed data. *p* < 0.05 was considered statistically significant. Transcriptomic and enrichment thresholds are specified in the corresponding methods above.

## Supplemental information

Table S1. Differential gene expression analysis of mouse tracheal allografts on day 7 versus syngeneic controls, related to Figure 1

Table S2. Differential gene expression analysis of mouse tracheal allografts on day 28 versus syngeneic controls, related to Figure 1

Table S3. Differential gene expression analysis of TGF-β1-induced MRC-5 myofibroblasts versus untreated fibroblasts in GSE225159, related to Figure 2

Table S4. Differential gene expression analysis of TGF-β1-treated HSAECs versus DMSO vehicle controls, related to Figure 5

Table S5. Differential gene expression analysis of HSAECs treated with TGF-β1 plus EMD638683 versus TGF-β1 alone, related to Figure 5

Data S1. Source images and data supporting histological, western blot, RT-qPCR, and transcriptomic analyses, including volcano plots, enrichment bubble plots, and heatmaps, related to Figures 1–6
